# Biogeography of Tick-Borne Bhanja Virus (*Bunyaviridae*) in Europe

**DOI:** 10.1155/2009/372691

**Published:** 2010-02-16

**Authors:** Zdenek Hubálek

**Affiliations:** ^1^Medical Zoology Laboratory, Institute of Vertebrate Biology ASCR, Klášterní 2, 69142 Valtice, Czech Republic; ^2^Institute of Vertebrate Biology, Academy of Sciences of the Czech Republic, v.v.i., Klášterní 8, 60365 Brno, Czech Republic

## Abstract

Bhanja virus (BHAV) is pathogenic for young domestic ruminants and also for humans, causing fever and affections of the central nervous system. This generally neglected arbovirus of the family *Bunyaviridae* is transmitted by metastriate ticks of the genera *Haemaphysalis, Dermacentor, Hyalomma, Rhipicephalus, Boophilus*, and *Amblyomma*. Geographic distribution of BHAV covers southern and Central Asia, Africa, and southern (partially also central) Europe. Comparative biogeographic study of eight known natural foci of BHAV infections in Europe (in Italy, Croatia, Bulgaria, Slovakia) has revealed their common features. (1) submediterranean climatic pattern with dry growing season and wet mild winter (or microlimatically similar conditions, e.g., limestone karst areas in central Europe), (2) xerothermic woodland-grassland ecosystem, with plant alliances *Quercetalia pubescentis, Festucetalia valesiacae, and Brometalia erecti*, involving pastoral areas, (3) presence of at least one of the tick species *Haemaphysalis punctata, Dermacentor marginatus, Rhipicephalus bursa*, and/or *Hyalomma marginatum*, and (4) presence of ≥60% of the 180 BHAV bioindicator (157 plant, 4 ixodid tick, and 19 vertebrate spp.). On that basis, Greece, France (southern, including Corsica), Albania, Spain, Hungary, European Turkey, Ukraine (southern), Switzerland (southern), Austria (southeastern), Germany (southern), Moldova, and European Russia (southern) have been predicted as additional European regions where BHAV might occur.

## 1. Introduction

**Figure 1:**
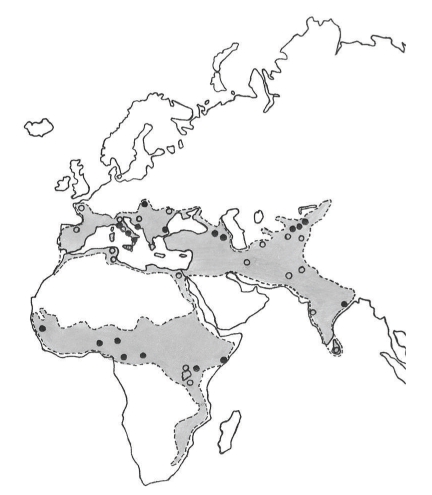
Bhanja virus distribution. Black points: virus isolation; circles: antibodies to BHAV present in vertebrates.

**Figure 2:**
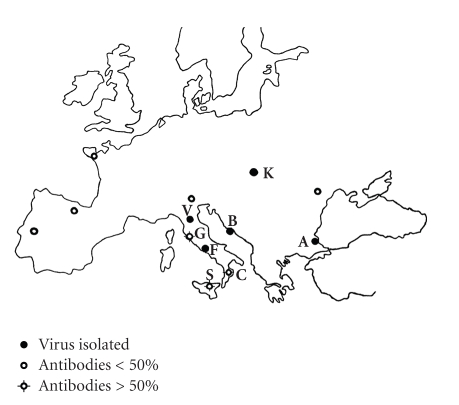
Bhanja virus in Europe at the time of the study start: dot: BHAV isolated; circle with four short lines: antibody positivity ≥50% of domestic ruminants; circle: seropositivity <50%. The letters indicate the selected foci; for their abbreviations see Section 2.

Bhanja virus (BHAV) is, together with two African tick-borne viruses Kismayo [1] and Forecariah [2], a member of Bhanja group (family *Bunyaviridae*) that has not yet been assigned to a recognized genus [3]. The virus was first isolated from the tick *Haemaphysalis intermedia* collected from a paralyzed goat in Bhanjanagar (district Ganjam, Orissa State, India) in 1954 [4]. The known geographic distribution of BHAV [5] involves southern and central Asia, Africa, and southern (partially central) Europe (Figure 1). In Europe (Figure 2), BHAV has so far been isolated in Italy [6], Croatia [7], Bulgaria [8], Slovakia [9], Romania [10], and Portugal (as Palma virus) [11].

The virus is transmitted by metastriate ixodid ticks: in addition to *H. intermedia, *it was also isolated from *Haemaphysalis punctata, H. sulcata, Dermacentor marginatus, Hyalomma marginatum, H. detritum, H. dromedarii, H. truncatum, H. asiaticum, Rhipicephalus bursa, R. appendiculatus, Boophilus decoloratus, B. annulatus, B. geigyi,* and* Amblyomma variegatum.*

Vertebrate hosts of BHAV are sheep, goat, cattle, African hedgehog *Atelerix albiventris, *and African ground squirrel *Xerus erythropus.* The virus does not usually cause apparent infection in adult animals but is pathogenic for young ruminants (lamb, kid, calf) causing fever and symptoms indicating the CNS affection [12–16]. Several cases of BHAV febrile illness have been described in humans, with symptoms including photophobia, vomiting, meningoencephalitis, and pareses [17–19]. Experimental encephalitis was produced in another primate species—rhesus monkey [20].

Modern predictive methods for potential geographic distribution of organisms, for example, of ixodid ticks or pathogens they transmit, are usually based on “remote sensing” satellite imagery combined with the use of geographic information system [21]. In this contribution, another approach is presented that is based on a “close (ground) sensing” of data. The procedure is characterized by comparing known natural foci of BHAV within Europe to extract their typical features and select those characters that can be used as factors predicting potential presence of the virus in other geographic areas [22].

## 2. Materials and Methodology

### 2.1. Description of Natural Foci of Bhanja Virus

**Figure 3:**
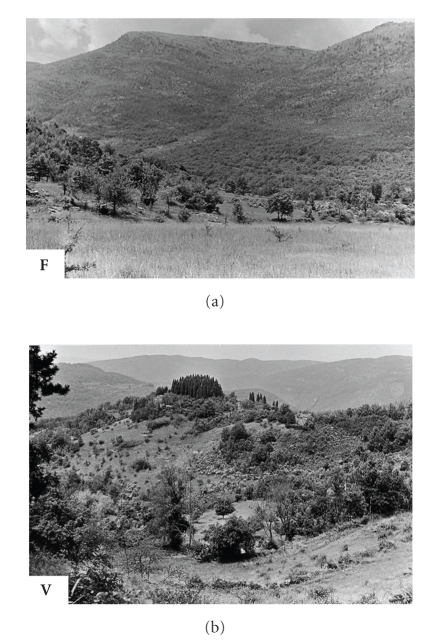
Natural focus of Bhanja virus infections in central Italy (Fondi-Campodimele, **F**) and northern central Italy (Toscana-Vaglia, **V**).

**Figure 4:**
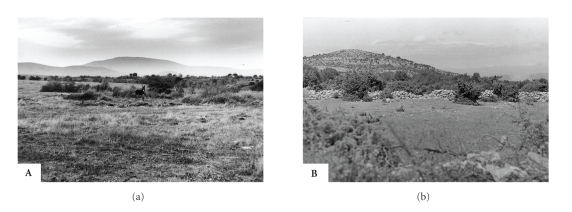
Natural focus of Bhanja virus infections in southeastern Bulgaria (Akhtopol, **A**) and Croatia (island of Brač, **B**).

**Figure 5:**
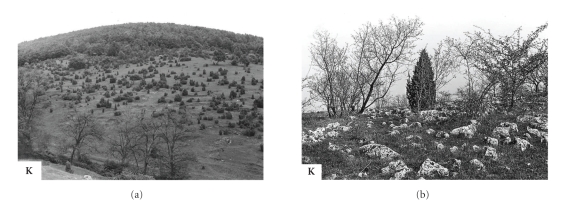
Natural focus of Bhanja virus infections in southeastern Slovakia (Slovak Karst at Kečovo, **K**).

Eight natural foci of BHAV in Europe were selected (Figure 2) being defined as those areas where the virus was either isolated from ixodid ticks or its presence indicated by a very high seroprevalence rate (≥50%) among local domestic ruminants in different years. The chosen areas include the following:

- **S**: Sicily around Catania and Madonie, Italy [23];
- **C**: Cosenza province, Calabria region, southern continental Italy [24];
- **F**: Fondi area—Monti Ausoni, Latina province, central Italy—Figure 3 [6];
- **G**: Grosseto province, Tuscany, central Italy [25];
- **V**: Vaglia, Florence province, Tuscany, northern central Italy—Figure 3 [26];
- **B**: Brač island, central Dalmatia, Croatia—Figure 4 [7, 27];
- **A**: Akhtopol and Akhtos, Burgas district, southeast Bulgaria—Figure 4 [8];
- **K**: Slovak Karst, Rožňava district, eastern Slovakia—Figure 5 [9].

Complex description of the natural foci has been based on physico-geographic characterization (for a list of the characters, see Supplementary Material available online at doi:10.1155/2009/372691): topography (40 characters), geology and pedology (47 characters), climate (39 characters), and gross vegetation (15 characters). The aim has been to search for features in common among the eight areas. Description of biota focused on terrestrial vascular plants (*Tracheophyta*), ixodid ticks (*Ixodidae*—Table 1) and vertebrates (*Vertebrata*), and based on checklists of species present in the eight European areas: a total of 2517 spp. terrestrial plants, 28 spp. ixodid ticks (Table 1) and 96 mammalian, 172 avian, and 40 reptilian spp. were included, each species occurring in at least one of the areas. A great number of resources were utilized for the survey: *Flora Europaea *[28–32] plus national and regional floral checklists, monographs [33–40] for geographic distribution of ixodid ticks, and an extensive collection of literature for vertebrates [22].

### 2.2. Numerical Comparative Analysis of the Foci

The data recorded as + or (+) were evaluated as the presence, - as the absence, while the variables with a doubtfull occurrence (?) in at least one of the two areas compared pairwise were omitted from the similarity calculations of the particular pair of areas. Numerical comparative pairwise analysis among the BHAV foci was carried out using the Jaccard similarity coefficient for binary (presence-absence) qualitative data, and Euclidean distance for normalized quantitative climatic data [41]. Matrices of both the similarity coefficient and Euclidean distance values were then subjected to the average linkage UPGMA cluster analysis [42], and the results were expressed as dendrograms of relationships (similarity) among the areas.

### 2.3. Selection of Bioindicators

The procedure for selection of “bioindicators” of Bhanja virus (i.e., those species indicating potential presence of BHAV in an area) consisted in the following steps at scanning the checklists of biota (i.e., terrestrial flora, ixodid, and vertebrate faunas): 

(1) “constant species” were considered those species present in all (8) or nearly all (6-7) BHAV areas; 

(2) “bioindicators” were then selected as those constant species having a relatively narrow ecological amplitude and a restricted distribution over Europe (i.e., ubiquitous and/or generalist/euryoic species were omitted).

## 3. Results

### 3.1. Physiographic, Climatic, and Biotic Similarity of the European Foci of Bhanja Virus

**Figure 6:**
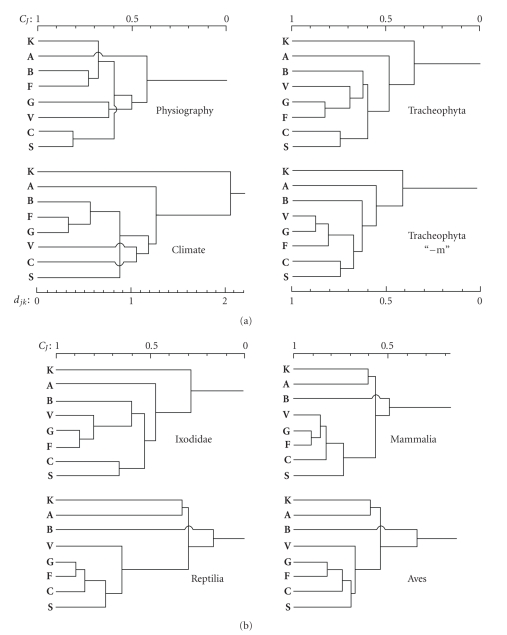
Dendrograms of similarity of the eight Bhanja virus areas in Europe, based on Jaccard coefficient (*C*_*J*_) or Euclidean distance (*d*_*jk*_—climate), and cluster analysis of the values. (a) Physiographic, climatic, and floral similarity; “−m,” Eumediterranean plus maritime littoral plant species were omitted (b) Faunal (ixodid and vertebrate) similarity.

The results of topographical, geological, pedological, and gross vegetational characters (102 in total) revealed a great similarity between the foci **C** and **S** on the one hand, among **B**, **F,** and **K** (all three are karst areas) on the other, while only a partial similarity between **V** and **G** and a marked dissimilarity of **A** (Figure 6).

**Figure 7:**
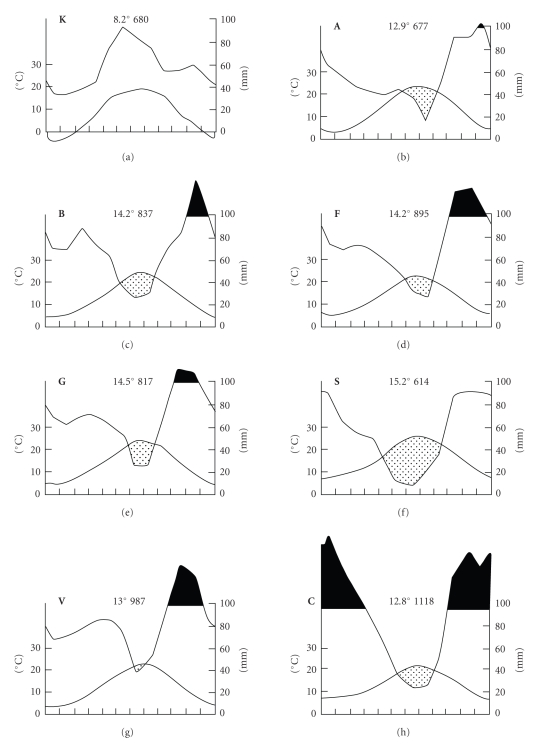
Climate characteristics of the eight Bhanja virus areas in Europe: climadiagrams sensu Walter (monthly course of mean air temperature and precipitation; mean annual temperature and mean total precipitation).

Figure 7 presents climadiagrams of annual course of temperature and precipitation, while Figure 6(a) shows the results of cluster analysis of the eight BHAV natural foci based on 39 climatic variables. The latter analysis indicates marked similarity among **F**, **G,** and **B** in their climate; other Italian foci (**S**, **V**, **C**) are also related, and Bulgarian **A** as well. On the other hand, Slovak **K** is considerably different from all other foci in its climate.

Cluster analysis of terrestrial flora among the foci resulted in a group consisting of three Central-Italian foci (**F**,** G**,** V**), related floristically to **B**, South-Italian foci (**C**,** S**) formed another group, while Bulgarian **A** is less similar, and Slovak **K** the least related focus (Figure 6). When the plant species restricted to Eumediterranean and littoral zone (“-m”) were excluded from the set, the result was slightly different: Central-Italian foci were floristically more similar to South-Italian foci than to Croatian **B**. Again, **A** and **K** deviated, the latter more than the former. The topology of dendrograms of avifaunal and herpetofaunal similarity of the foci was identical, and the dendrogram based on mammalian faunas deviated only negligibly (Figure 6). All these three dendrograms showed the formation of three clusters of foci: (a) all Italian, (b) Croatian **B**, (c) **A** and **K**. On the other hand, ixodid faunas yielded a dendrogram (Figure 6(b)) very similar to that based on *Tracheophyta*, with formation of two clusters and two singletons: (a) Central-Italian foci (**F**,** G**,** V**) and Croatian **B**; (b) South-Italian foci (**C**,** S**), (c) Bulgarian **A**, and (d) Slovak **K**. It means that there is a notable congruence between the similarity of foci based on fauna of *Ixodidae *(a number of them being vectors of BHAV) and that based on terrestrial flora, whereas vertebrate faunas yielded a different pattern [22].

Calculations of the congruence among the biotic and abiotic components of the foci by Pearson correlation coefficient [42] revealed that ixodid fauna reflects best the terrestrial flora and also climate, much less correlates with vertebrate fauna, whereas physiographic features do not correlate with the other components. In other words, there is an evident, statistically significant relation between climate, terrestrial vascular plants, and ixodid ticks, where the climate is the ultimate causative factor; vegetation reflects these abiotic weather conditions and affects (shelter, etc.) or indicates the formation of tick synusies, while much less closely that of vertebrate communities.

### 3.2. Common Biogeographic Features of the Natural Foci

A number of biogeographic features in common was found among the natural foci of BHAV in Europe. For instance, mean altitude is 200–850 (avg. 530) m a.s.l. The relief is undulating to hilly (or low mountainous), with presence of terraces, plateaus, or peneplains. Hydrographic net is sparse, combined with largely dry soils. Marine transgression of the area usually occurred during the Triassic, Lower Jurassic, Upper Cretaceous (Senonian), Oligocene, and partly Mid-Miocene periods. Mesozoic (Cretaceous or Triassic) or Tertiary (largely Pliocene, Eocene) deposits occur. Sedimentary rocks (in contrast to metamorphic and igneous rocks) prevail; consequently carbonate minerals (marls, marly, or clayey shales, sandstones, conglomerates, clays, partly limestone) are much more usual than silicates. Frequent soil types are rendzinas, terrae calcis (e.g., terra rossa), and illimerized and brown forest soils; the soil is liable to erosion and drought. An agrarian (pastoral) type of landscape is typical. Climate has the Mediterranean pattern, with peak rainfall in the winter (Figures 3 and 4), and a dry, hot summer period or, in central Europe (**K**, Slovak Karst—Figure 5), there are extrazonal xerothermic habitats on karstified limestone approaching meso- and microclimatically those in southern Europe. Mean annual precipitation is 600–1100 mm, air temperature 8–15°C (January 4°C, July 23°C); hydrothermic coefficients indicate climate corresponding to the ecotone between steppe and forest ecosystems. Dominating vegetation type is mosaic “woodland-grassland (forest-steppe) ecosystem,” consisting largely of thermophilic, broad-leaved deciduous woods (*Quercetalia pubescentis) *or pubescent oak bush, and xerothermic meadows with pastures (*Festucetalia valesiacae, Brometalia erecti, Onopordetalia acanthii, Prunetalia, Origanetalia vulgaris*). Typical for the BHAV foci is the colin vegetational belt with trees like *Quercus pubescens, Fraxinus ornus, Ostrya carpinifolia, Sorbus torminalis, Prunus mahaleb, * and so forth; vineyards and thermophilic orchards are common. Natural foci of BHAV in Europe do not occur in the Eumediterranean *Oleo-Ceratonion* and *Quercion ilicis* zones. The foci are exclusively of boskematic type [43], that is, associated with grazing domestic ruminants (largely sheep and goats) as main hosts of local ixodid vector ticks. Characteristic taxa of wild vertebrates are those living in relatively open, dry, and warm habitats—for instance, species of the avian genera *Circaetus, Falco, Alectoris, Caprimulgus, Upupa, Calandrella, Lullula, Oenanthe, Monticola, Lanius*.

### 3.3. Biogeographic Predictors of the Natural Foci

Four groups of principal biogeographic predictors for BHAV potential areas in Europe have been established, based on comparative analysis of the eight European foci:

(1) submediterranean climatic pattern with dry growing season and wet mild winter (or mesoclimatically similar, e.g., karstic conditions in Central Europe),

(2) xerothermic woodland-grassland ecosystem, with plant alliances *Quercetalia pubescentis, Festucetalia valesiacae*, and *Brometalia erecti*, involving pastoral areas with regular grazing of (small) ruminants, on undulating to hilly relief,

(3) at least one of the metastriate tick species *Haemaphysalis punctata, Dermacentor marginatus, Rhipicephalus bursa *and *Hyalomma marginatum.*

(4) Presence of 60% or more of the 180 (157 plant, 4 tick, 19 vertebrate) spp. (or species aggregations) selected bioindicators.

### 3.4. Bioindicators

As a result of the comparative analysis, 157 plant, 6 mammalian, 9 avian, 4 reptilian, and 4 ixodid spp. (or species aggregations) were selected as bioindicators.

**Figure 8:**
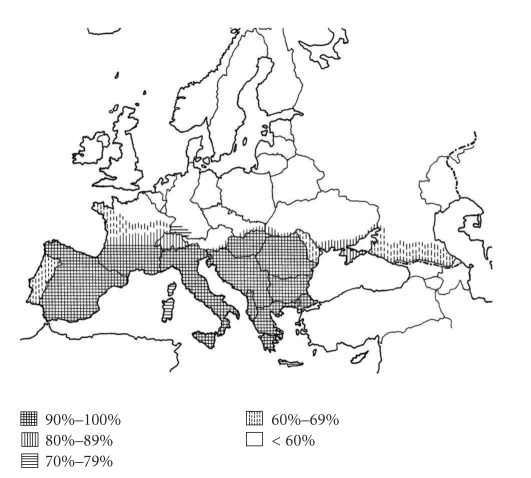
Prediction of potential Bhanja virus area in Europe, based on proportion of the bioindicator species present in particular countries and areas.

Table 2 shows suggested bioindicators of BHAV natural foci in Europe. Predicted countries and areas in Europe where BHAV could potentially occur have been based on the presence of a sufficiently high (≥60%) proportion of species of the bioindicator set (Figure 8). The countries (areas) with predicted occurrence of Bhanja virus include the following (Table 3): Italy, former Yugoslavia (present Slovenia, Croatia, Serbia, Monte Negro, Macedonia, Bosnia), Bulgaria, Greece, France (southern), Albania, Romania, Spain, Hungary, European part of Turkey, Ukraine (southern), Slovakia (southern), Switzerland (Tessin), Austria (southeastern), Corsica, Sardinia, Germany (southern), Moldova, European Russia (southern), Portugal, and possibly Crete. On the other hand, European countries with improbable occurrence of Bhanja virus are Belgium, Czechland (Czech Republic), Poland, Great Britain, The Netherlands, Belarus, Sweden, Denmark, Ireland, Lithuania, Norway, Latvia, Estonia, Finland, and Iceland.

The geographic distributional centre of the suggested BHAV bioindicators is largely the East-Mediterranean region, usually with links to Asia Minor and Transcaucasus. Further, the number of zooindicators is much lower than that of phytoindicators, though the proportions are identical (6% of the total number of species recorded). Ecological index sensu Ellenberg [44] yielded for 70 spp. of the BHAV plant indicators an average score of “774–383,” with the meaning “a xerothermic plant growing on insolated places of suboceanic climate and alkaline (often calcareous), largely nitrogen-deficient soil.” The phytoindicator species lying very close by their ecological indices to this average index value have been, for example, *Quercus pubescens, Cerastium brachypetalum, Arabis turrita, Conringia orientalis, Amelanchier ovalis, Prunus mahaleb, Lathyrus latifolius, L. hirsutus, Coronilla emerus, Dictamnus albus*.* Acer monspessulanum, Helianthemum canum, Cornus mas, Scandix pecten-veneris*, *Galium tricornutum, Asperula cynanchica, A. arvensis, Teucrium chamaedrys, Stachys germanica, S. recta, Legousia speculum-veneris, Chondrilla juncea, Lactuca viminea, Filago vulgaris, Hieracium piloselloides, Muscari comosum, Melica ciliata, Agropyron intermedium, Phleum paniculatum*, and* Himantoglossum hircinum.*

## 4. Discussion

Eight areas in Europe were selected for comparison and called natural foci. In general, a natural focus (nidus) of a disease is an area where the agent circulates in an ecosystem between vertebrates and haematophagous arthropod vectors for many years [43, 45]. In a natural focus, the agent should be therefore found repeatedly (in consecutive years) either directly (by isolation from arhropod vectors or vertebrate hosts) or indirectly, by detection of antibodies in vertebrates. All eight areas selected in this study fulfil this criterion of a natural focus. For instance, even in the most “deviating” and northernmost area, Slovak Karst, antibodies to BHAV were first detected in goats and sheep in 1976 [46], then repeatedly in the years 1982 and 1983 [22], and BHAV was isolated from local *Dermacentor marginatus* ticks in 1987 [9].

In general, groups of species or whole communities are often more reliable as bioindicators than individual species. Therefore only the presence of substantial proportion of the proposed set of indicator species can have a sufficient predictive power. In this study, the cut-off level was established tentatively at 60%.

A unique opportunity to verify the set of predictors of BHAV occurrence established in this study is a comparison of two close and in many respects very similar Dalmatian islands Brač and Hvar (Croatia). There is, namely, a known, potent natural focus of BHAV infections on Brač (**B** in this study), whereas no known BHAV focus on Hvar. Vesenjak-Hirjan et al. [27] detected antibodies to BHAV in 31.5% of inhabitants (875 examined) on Brač but only in 1.0% (512 examined) on Hvar, though antibodies to, for example, sandfly fever were distributed homogeneously among inhabitants of both islands: 62.1% and 59.4%, respectively. The authors noted that “the underlying reasons for such a great difference on two neighbouring islands have not as yet been explained.” However, the suggested set of predictors of BHAV foci does enable clear differentiation of both islands in the following crucial factors: (1) plant community *Ostryo-Carpinion adriaticum* (order *Quercetalia pubescentis*), occurring in the central elevated part of Brač, does not appear on Hvar; (2) very limited numbers of domestic ruminants graze on Hvar (typical extensive pastures are completely lacking) contrasting to their large numbers (even for centuries) on Brač; (3) population density of BHAV vector ticks (*Haemaphysalis punctata, Dermacentor marginatus*) is (consequently) much lower on Hvar than on Brač. It is therefore probable that this predictive approach could well be used to test the probability of potential BHAV occurrence even in individual districts within a particular country.

The possibility of indication of natural foci of tick-borne encephalitis (TBE) by the presence of specific plant communities was proposed by parasitologists and botanists more than four decades ago [47–49]. However, *Ixodes ricinus*, the vector of TBE, is a generalist (euryoic) tick species with a very wide ecological valence and therefore much less amenable to predictive analysis. On the other hand, prediction is relatively easier in the specialist (stenoic) species like some metastriate ticks, including vectors of BHAV, having a rather narrow ecological valence. These species are generally bound to certain specific habitats and the analysis of their distribution can thus be more straightforward and effective.

The present paper shows that a “close/ground sensing,” although not so sophisticated, is an alternative to remote sensing and applicable for prediction of geographic distribution of ixodid ticks and pathogens they transmit. A prerequisite for this alternative approach is existence of corresponding databases and/or checklists of abiotic and biotic variables in respective areas or a complex field survey.

An anonymous reviewer of this manuscript suggested that BHAV infection could have followed humans with their domestic animals (*Bos indicus*) infested with one-host vector ticks such as *Boophilus* spp. from Asia to Africa and a mixture of African and Asian cattle (*Bos taurus*) into the Mediterranean. Additionally, primaeval movements of tick-infested sheep and goats from Asia to Africa and Europe could play an important role in the dispersion of BHAV.

## Supplementary Material

List of physiographic, climatic, and vegetational characteristics used for numerical comparison of the eight Bhanja virus areas in Europe.

## Figures and Tables

**Table 1:** Checklist of ixodid fauna in eight European BHAV areas (example of one of the three checklists of biota, *viz Ixodidae*, *Vertebrata*, and *Tracheophyta*).

Tick species	**K**	**A**	**B**	**F**	**C**	**S**	**V**	**G**
* Ixodes trianguliceps *Birula	+	−	−	−	−	−	−	−
* Ixodes arboricola *Schulze & Schlottke	+	(+)	−	−	−	−	−	−
* Ixodes crenulatus *Koch	+	?	−	+	−	−	+	(+)
* Ixodes hexagonus* Leach	+	(+)	?	+	?	+	+	(+)
* Ixodes frontalis *Panzer	−	−	−	+	−	−	+	(+)
* Ixodes simplex *Neumann	(+)	+	−	−	−	−	−	−
* Ixodes vespertilionis *Koch	+	+	+	+	−	−	+	+
* Ixodes acuminatus *Neumann	−	−	−	(+)	−	−	−	+
* Ixodes gibbosus *Nuttall	−	−	+	(+)	−	−	−	−
* Ixodes ricinus *(L.)	+	+	−	+	+	+	+	+
* Ixodes laguri *Olenev	+	+	−	−	−	−	−	−
* Dermacentor marginatus *(Sulzer)	+	+	+	+	+	+	+	+
* Haemaphysalis punctata *Canestrini & Fanzago	+	+	+	+	+	+	+	+
* Haemaphysalis sulcata *Canestrini & Fanzago	−	+	+	+	?	+	+	+
* Haemaphysalis erinacei *Pavesi	−	+	−	−	−	−	−	−
* Haemaphysalis inermis *Birula	−	+	−	−	−	−	−	−
* Haemaphysalis parva *Neumann	−	+	+	+	+	−	−	+
* Rhipicephalus bursa *Canestrini & Fanzago	−	+	+	+	+	+	+	+
* Rhipicephalus sanguineus *(Latreille)	−	+	+	+	+	+	+	+
* Rhipicephalus turanicus *Pomerancev	−	+	−	−	−	−	−	−
* Boophilus annulatus *Say	−	+	(+)	+	+	+	+	+
* Hyalomma marginatum *Koch	−	+	+	+	+	+	+	+
* Hyalomma aegyptium *(L.)	−	+	−	+	−	−	−	−
* Hyalomma detritum *Schulze	−	(+)	−	−	–	+	−	−
* Hyalomma scupense *Schulze	−	+	+	−	−	−	−	−
* Hyalomma lusitanicum *Koch	−	−	−	−	+	+	−	−
* Hyalomma excavatum *(Koch)	−	−	−	−	−	+	−	−
* Amblyomma variegatum *(Fabricius)	−	−	−	−	−	(+)	−	−

The species is –, absent; +, present; (+), rare; ?, dubious in the area. For letter abbreviations of areas, see Section 2.

**Table 2:** Bioindicator species of the potential Bhanja virus occurrence in Europe.

ACARINA Hard ticks	4 spp.
*Ixodidae*	*Haemaphysalis punctata, Dermacentor marginatus, Hyalomma marginatum, Rhipicephalus bursa*

VERTEBRATA Mammals, birds, reptiles	19 spp.

*Rhinolophidae*	*Rhinolophus ferrumequinum, R. euryale*

*Vespertilionidae*	*Myotis emarginatus, M. blythi, M. capaccinii, Miniopterus schreibersii*

*Accipitridae*	*Circaetus gallicus*

*Phasianidae*	*Alectoris graeca*

*Strigidae*	*Otus scops*

*Alaudidae*	*Calandrella cinerea*

*Turdidae*	*Monticola saxatilis, Oenanthe hispanica*

*Laniidae*	*Lanius senator*

*Emberizidae*	*Emberiza cirlus, E. cia*

*Lacertidae*	*Lacerta muralis* s.l. (incl.* L. melisellensis, L. sicula*)*, L. viridis* s.l. (incl.* L. trilineata*)

*Colubridae*	*Coluber gemonensis* s.l. (incl*. C. jugularis, C. viridiflavus*)*, Elaphe longissima *

TRACHEOPHYTA Vascular plants	157 spp.

*Aspleniaceae*	*Ceterach officinarum*

*Corylaceae*	*Ostrya carpinifolia*

*Fagaceae*	*Quercus cerris, Q. pubescens*

*Ulmaceae*	*Celtis australis*

*Santalaceae*	*Thesium linophyllon*

*Caryophyllaceae*	*Minuartia hybrida, Cerastium brachypetalum, Silene italica, S. viridiflora, S. gallica, S. conica, Petrorhagia prolifera*

*Ranunculaceae*	*Clematis vitalba*

*Cruciferae*	*Arabis turrita, A. auriculata, Eruca vesicaria, Calepina irregularis, Lepidum graminifolium, Hornungia petraea, Thlaspi perfoliatum, Conringia orientalis*

*Saxifragaceae*	*Saxifraga bulbifera*

*Rosaceae*	*Rubus ulmifolius, R. canescens, Rosa gallica, Potentilla recta, Sorbus domestica, S. torminalis, Amelanchier ovalis, Prunus mahaleb*

*Leguminoseae*	*Colutea arborescens, Vicia grandiflora, V. tenuifolia, V. narbonensis, V. lutea, Lathyrus aphaca, L. latifolius, L. sphaericus, L. nissolia, L. hirsutus, Ononis pusilla, Trigonella gladiata, T. monspeliaca, Medicago arabica, Trifolium subterraneum, T. scabrum, Dorycnium pentaphyllum, Coronilla emerus, Hippocrepis comosa*

*Geraniaceae*	*Geranium rotundifolium, G. purpureum, Erodium ciconium*

*Linaceae*	*Linum tenuifolium, L. trigynum*

*Euphorbiaceae*	*Euphorbia platyphyllos*

*Rutaceae*	*Dictamnus albus*

*Aceraceae*	*Acer monspessulanum*

*Malvaceae*	*Althaea hirsuta, A. cannabina*

*Thymelaeaceae*	*Thymelaea passerina*

*Cistaceae*	*Cistus incanus, C. salvifolius, Fumana procumbens, Helianthemum canum, Tuberaria guttata*

*Cornaceae*	*Cornus mas*

*Umbelliferae*	*Eryngium campestre, Bupleurum praealtum, Tordylium maximum, Torilis arvensis, Turgenia latifolia, Scandix pecten-veneris, Smyrnium perfoliatum, Trinia glauca *

*Oleaceae*	*Ligustrum vulgare, Fraxinus ornus*

*Rubiaceae*	*Galium tricornutum, Asperula arvensis, A. cynanchica*

*Convolvulaceae*	*Convolvulus cantabrica*

*Boraginaceae*	*Lithospermum purpureocaeruleum, Anchusa azurea, Heliotropium europaeum*

*Labiateae*	*Teucrium chamaedrys, T. montanum, Prunella laciniata, Stachys germanica, S. recta, Calamintha sylvatica, C. nepeta*

*Solanaceae*	*Solanum luteum*

*Scrophulariaceae*	*Verbascum phlomoides, Scrophularia canina, Antirrhinum orontium, Kickxia elatine, K. (Linaria) spuria*

*Globulariaceae*	*Globularia punctata*

*Orobanchaceae*	*Orobanche ramosa, O. purpurea, O. gracilis, O. alba, O. minor, O. lutea*

*Valerianaceae*	*Valerianella coronata*

*Dipsacaceae*	*Cephalaria transsylvanica*

*Campanulaceae*	*Campanula rapunculus*, *Legousia speculum-veneris *

*Compositae*	*Bombycilaena (Micropus) erecta, Filago vulgaris, Inula conyza, Echinops ritro, Crupina vulgaris, Centaurea triumfetti, C. solstitialis, C. calcitrapa, Carthamus lanatus, Carduus nutans*,* Scorzonera laciniata, Chondrilla juncea, Lactuca viminea, L. saligna, Hieracium piloselloides *

*Liliaceae*	*Gagea arvensis, Ornithogalum umbellatum, O. comosum, O. pyramidale *s.l. (incl.* O. narbonense*)*, Scilla autumnalis, Muscari comosum*.*, M. racemosum, M. botryoides, Allium sphaerocephalon, A. flavum, A. paniculatum, Asparagus acutifolius, A. tenuifolius, Ruscus aculeatus *

*Dioscoreaceae*	*Tamus communis*

*Gramineae*	*Sclerochloa dura, Melica ciliata, Bromus erectus, Agropyron intermedium, Aira elegantissima, Phleum paniculatum, Stipa pennata, S. pulcherrima, S. bromoides, Cleistogenes serotina, Cynodon dactylon, Tragus racemosus, Botriochloa ischaemum*

*Cyperaceae*	*Carex halleriana*

*Orchidaceae*	*Limodorum abortivum, Orchis tridentata, Himantoglossum hircinum, Anacamptis pyramidalis, Ophrys sphecodes*

**Table 3:** Bhanja virus—prediction of occurrence in European countries.

Country (area)	Proportion of the 180 bioindicator spp. (%)	BHAV presence
Italy	100	demonstrated
Yugoslavia (former)	100	demonstrated
Bulgaria	100	demonstrated
Greece	97	probable
France (south)	97	probable
Sicily	94	probable
Albania	94	probable
Romania	94	demonstrated
Spain	92	probable
Hungary	91	probable
European Turkey	91	probable
Ukraine (southern)	89	probable
Slovakia (southern)	84	demonstrated
Switzerland (Tessin)	83	probable
Austria (southeast)	82	probable
Corsica	79	probable
Sardinia	77	probable
Germany (southern)	74	probable
Moldova	69	probable
European Russia (south)	68	probable
Portugal	66	demonstrated
Crete	59	possible
Belgium	51	improbable
Czechland	47	improbable
Poland	46	improbable
Great Britain	43	improbable
The Netherlands	33	improbable
Belarus	26	improbable
Sweden	18	improbable
Denmark	17	improbable
Ireland	14	improbable
Lithuania	8	improbable
Norway	7	improbable
Latvia	6	improbable
Estonia	6	improbable
Finland	3	improbable
Iceland	1	improbable
