# Supplementary material for: Biogeography of Tick-Borne Bhanja Virus (Bunyaviridae) in Europe
Source: Interdiscip Perspect Infect Dis. 2010 Feb 16;2009:372691. doi: 10.1155/2009/372691 (PMC2825549; doi:10.1155/2009/372691)
Supplement: Supplementary file 1 — List of physiographic, climatic, and vegetational characteristics used for numerical comparison of the eight Bhanja virus areas in Europe. [file 372691.f1.pdf]

List of physiographic, climatic, and vegetational characteristics used for numerical comparison of Bhanja virus areas in Europe.

Topography (40 qualitative characters)

- Altitude (m a.s.l.): minimum – maximum – mean.
- Relief classes: flat – undulating to rough – hilly – mountainous.
- Mean slope steepness (grades): minimum – maximum – average.
- Presence of: cliffs or scarps – canyons or ravines – significant tectonic faults – colluvial cones or footslope deposits – man-made stony walls and piles – fluvial or abrasion terraces - plateaus or peneplains – hums – karren fields – dolines (sink holes) – uvalas – poljes or semipoljes – blind or dry valleys – caverns – jamas (deep pits) – ponors (swallow holes) – springs or karst springs – vruljas (submarine springs) – lakes or temporary lakes – stable surface streams – periodical surface streams – pseudokarst.

Geology and soil (47 qualitative characters):

- Geological formations: Palaeozoic (Carboniferous) – Lower Triassic (Werfenian) – Middle Triassic (Ladinian) – Upper Triassic (Keuper) – Lower Jurassic (Lias) – Middle Jurassic (Dogger) – Upper Jurassic (Malm) – Lower Cretaceous – Upper Cretaceous (Senonian) – Palaeocene – Eocene – Oligocene – Miocene – Pliocene – Pleistocene – Holocene.
- Rocks and minerals: igneous rocks – sedimentary rocks – metamorphic rocks – silicates – carbonate minberals – Flysch – granite or granodiorite – diorite, rhyolite or dacite – gabbro or porphyry – biotite, amphibolite or serpentite – tuff, basalt or

trachyte – andesite, andesite tuffs or tuffite – gneiss or crystalline shales – phyllites or quartzites – limestone (calcite) – dolomite – marble or crystalline limestone – marls – marly or clayey shales – sandstone or sands (arenaceous deposits) – conglomerates or breccias – clays or argillaceous deposits – aggregates (gravel).

- Soil types: protorendzina – rendzina – terra rossa or terra fusca – illimerized soils – brown forest soil – yellow subtropical soil – smolnitza.
- Soil erosion.

Climate (39 quantitative characters):

- Mean air temperature: annual average – January – April – July – October – annual range.
- Mean number of: frosty days – icy days.
- Mean length of growing season with : 5 ºC – 10 ºC.
- Mean cloudiness (%).
- Mean precipitation: January – April – July – October.
- Percent of annual precipitation: Spring – Summer – Autumn – Winter – Warm season (April to September).
- Mean number of days with: snowfall – snow cover.
- Mean relative humidity of the air (%): January – April – July – October.
- Lang's rainfall factor.
- Minář's moisture index.
- DeMartonne's aridity index: January – April – July – October.
- Seljaninov's hydrothermic coeffcicient: April – July – October – growing season (April to Septemeber) number of days with coefficient 1.0 or less – 0.5 or less.

Gross vegetation (15 qualitative characters):

- Forests: coniferous or mixed – deciduous broad-leaved – evergreen broad-leaved.
- Scrub: maquis – garigue – pseudomaquis – šibljak (Karstbuschwald).
- Ecosystems: forest-steppe or open woodland – open grassland – extensive arable fields – vineyards plus orchards.
- Vegetation levels: planar – colin – submontan – montan (remark: subalpine and alpine levels were absent in all eight natural foci).
